# Physiological and molecular mechanisms of insect appendage regeneration

**DOI:** 10.1186/s13619-022-00156-1

**Published:** 2023-03-02

**Authors:** Jiru Zhong, Andi Jing, Shaojuan Zheng, Sheng Li, Xiaoshuai Zhang, Chonghua Ren

**Affiliations:** 1 Guangdong Provincial Key Laboratory of Insect Developmental Biology and Applied Technology, Guangzhou Key Laboratory of Insect Development Regulation and Application Research, Institute of Insect Science and Technology, School of Life Sciences, South China Normal University, Guangzhou, 510631 China; 2 Guangmeiyuan R&D Center, Guangdong Provincial Key Laboratory of Insect Developmental Biology and Applied Technology, South China Normal University, Meizhou, 514779 China

**Keywords:** Appendage regeneration, Physiological condition, Molecular mechanism, Signaling pathways, Epigenetics, Insects

## Abstract

Regeneration, as a fascinating scientific field, refers to the ability of animals replacing lost tissue or body parts. Many metazoan organisms have been reported with the regeneration phenomena, but showing evolutionarily variable abilities. As the most diverse metazoan taxon, hundreds of insects show strong appendage regeneration ability. The regeneration process and ability are dependent on many factors, including macroscopic physiological conditions and microscopic molecular mechanisms. This article reviews research progress on the physiological conditions and internal underlying mechanisms controlling appendage regeneration in insects.

## Background

Regeneration refers to the phenomenon that individual body part can be restored to its original structure after being amputated or damaged (Kerkut 1985). Studies of insect regeneration mainly focus on the appendage (including legs, antennae, imaginal disc) and organ regeneration, such as intestine (Mattila et al. 2005). In this review, we mainly focus on appendage regeneration rather than organ regeneration. All the insect appendages, highly complex structures composed of epidermis, muscles and nerves, can regenerate (Fox et al. 2020; French 1976). Because of the existence of exoskeleton, appendage regeneration is sheltered by this chitin shell, thus a complete appendage regeneration requires molting. In contrast to the mammal limited regeneration capacity, many insect appendages can be restored to their original tissue size and spatial organization after the molting process.

**Fig. 1:**
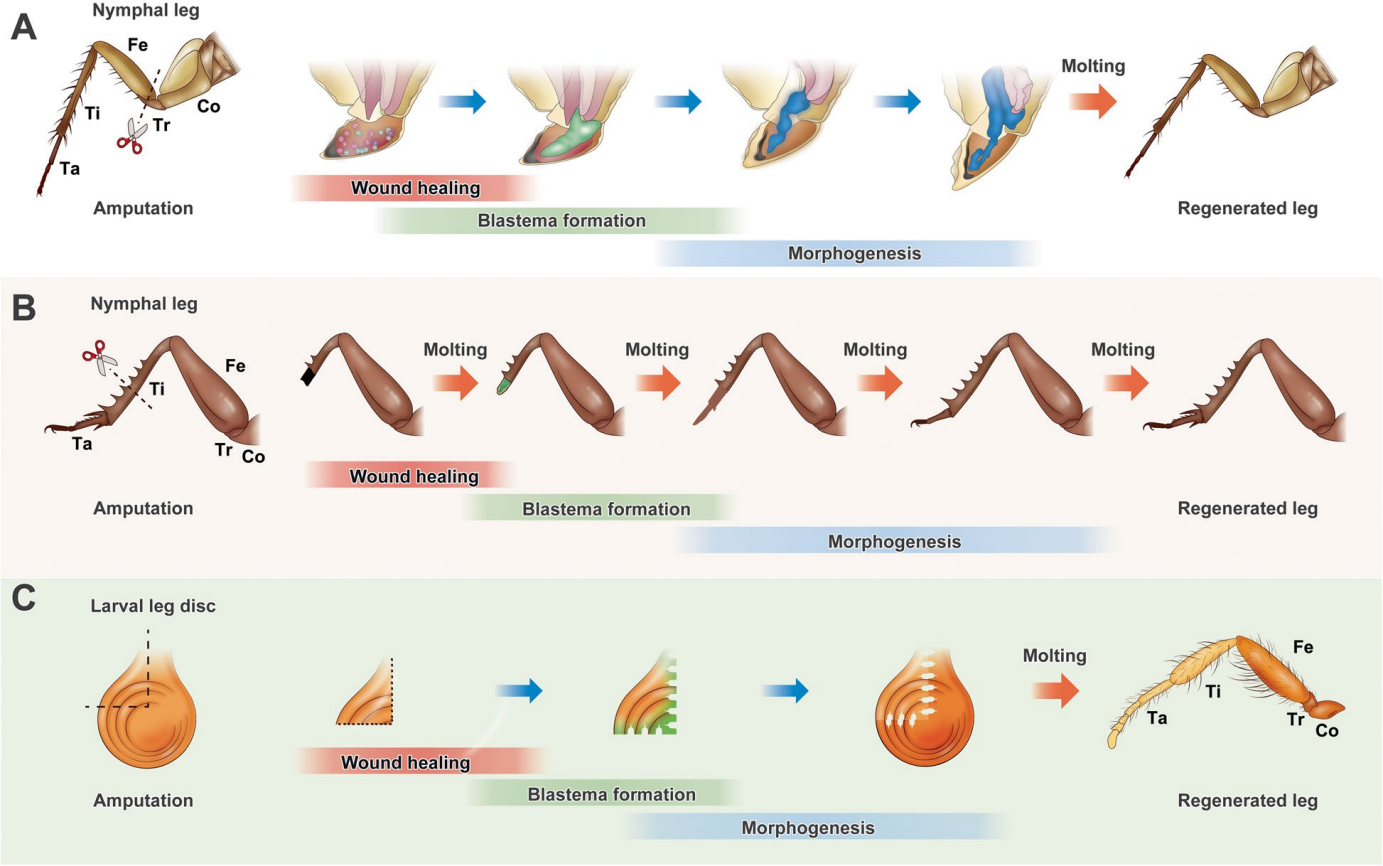
Diagram of appendage regeneration in insects. Following injury or amputation, appendage regeneration in nymphal legs (**A** and **B**) or larval imaginal discs (**C**) proceeds through a generally similar process involving three stages: wound healing, blastema formation, and morphogenesis (regenerative growth, differentiation, and patterning). **A** In some Blattaria and other orders of insects, when amputation is performed at a site proximal to the femur, a new leg can be regenerated under the exoskeleton after one molt. **B** In some insects of the order Orthoptera, regeneration mainly occurs at the distal site of the tibia. Four molting cycles are needed for the successful regeneration of missing tissue. **C** After the surgical amputation of the imaginal leg disc, the smaller fragment from the anterior dorsal quadrant regenerates the remainder of the disc, and a new leg can be regenerated after one molt. Co, coxa; Tr, trochanter; Fe, femur; Ti, tibia; Ta, tarsus

Up to now, many insects, including incompletely metamorphosed cockroaches, crickets, beetles and holometabolous fruit flies, have been described the phenomenon of appendage regeneration and served as excellent models for laying the foundation of appendage regeneration in insect. Cockroaches, such as the American cockroach *Periplaneta americana*, have been major subjects of research in the regeneration field since the first record of its leg regenerative phenomenon, and many well-known regenerative theories and concepts, such as intercalary regeneration, the polar coordinate model, and the boundary model, have been established in cockroaches (Anderson and French 1985; Bell and Adiyodi 1982; French et al. 1976; Meinhardt 1982) (Fig. 1A). Many molecular studies have also been performed in another familiar insect model that undergoes incomplete metamorphosis, *Gryllus bimaculatus* (Bando et al. 2018) (Fig. 1B). Given the well-established genetic resources available for the *Drosophila melanogaster* holometamorphosis model, great progress has been made in the study of regenerative mechanisms using imaginal discs (Belacortu and Paricio 2011) (Fig. 1C).

The process of insect limb regeneration can be generally divided into three stages: wound healing, blastema formation, and morphogenesis (regenerative growth, differentiation, and patterning) (Feleke et al. 2021; Zhou et al. 2021) (Fig. 1). In the wound healing stage, the muscles of the wounded organ generally contract around the site of the injury, and the wound is then sealed by a clot of hemolymph, which hardens to form a scab soon after amputation. Then, large numbers of hemocytes congregate underneath the scab (Day 1952). At the same time, the epidermal cells to either side of the wound become enlarged, and the nucleolus becomes more distinct. After wound healing, activation and mitotic activity of cells spread outward from the wound (Khan et al. 2016). Blastema formation is the key event in tissue regeneration. The regeneration extremely depends on the proliferation of blastema cells, a population of dedifferentiated cells that forms adjacent to the wound epidermis, in most cases. Depending on the forms of blastema formation, two models are proposed. The polar coordinate model proposed by Penzlin shows that cell division may only occur near the injury site or wound to form blastema (Penzlin 1963). Another model described by Bulliere shows that blastema formation involves the reshaping of the surrounding epidermis, rather than the division of cells near the wound area (Bullière and Bullière 1977). During the morphogenesis stage, these blastema cells are responsible for the regeneration and reorganization of the missing imaginal disc fragments or appendages, thereby restoring the damaged or missing parts, including muscles, nerves, tracheae, and epidermal tissue (Khan et al. 2016).

Regeneration in insects is controlled by physiological factors and internal molecular mechanisms that involve multiple signaling pathways and other regulatory strategies (Tables 1, 2). In this article, we mainly focus on the physiological factors and internal mechanisms controlling insect appendage regeneration and briefly introduce future directions in this field.

**Table 1:** Physiological conditions controlling regeneration

Physiological conditions	Regeneration diversity	
Developmental and life stages	regeneration loss in adult (metamorphotic insects)	regeneration remain in adult (ametabolous insects)
	malformed in adults when excised at larger instars	
	regeneration loss after critical point	
Degree of amputation	negative correlation between amputation degree and regeneration ability	
Species diversity	regeneration ability varies along with species diversity

**Table 2:** Internal mechanisms underlying regeneration

Internal factors		Regeneration process	Species and tissues	Regulatory network	References
Signaling pathways	ROS and MAPK	wound healing, regenerative growth	*D. melanogaster*: wing disc; *A. ypsilon:* wing disc	MAPK signalings can be activated by ROS	Bergantiños et al. 2010; Bosch et al. 2005; Mattila et al. 2005; Xu et al. 2021
	ECM	wound healing, blastema formation	*D. melanogaster*: leg disc; *T. castaneum*: larval leg	Mmps are induced by JNK signaling	McClure et al. 2008; Mitten et al. 2012
	JAK/STAT	blastema cell proliferation	*D. melanogaster:* wing disc; *G. bimaculatus*: nymphal leg	Induced by Toll-2 and ROS/JNK/P38/Upd cascade	Bando et al. 2013; La Fortezza et al. 2016; Santabárbara-Ruiz et al. 2015); Bando et al*.* 2022
	TGF-β	blastema formation and patterning	*G. bimaculatus*: nymphal leg; *P. americana*: nymphal leg	Interacts with Dachsous/Fat signaling and *dac* gene	Mito et al. 2002; Ishimaru et al. 2018; Li et al. 2018
	Hippo	blastema cell proliferation	*D. melanogaster*: wing disc; *G. bimaculatus*: nymphal leg	Activated by JNK signaling and Dachsous/Fat signaling	Grusche et al. 2011; Sun and Irvine 2011; Bando et al*.* 2018; Bando et al. 2009
	Wg/Wnt	initiation and patterning	*D. melanogaster*: wing and eye discs; *T. castaneum*: larval leg, antenna, maxilla; *G. bimaculatus*: nymphal leg	Activated by JNK signaling	Schubiger et al. 2010; Smith-Bolton et al. 2009; Sustar et al. 2011; Shah et al. 2011; Nakamura et al. 2008a, b; Nakamura et al. 2007
	Hedgehog	patterning and regenerative growth	*D. melanogaster:* leg disc; *H. axyridis*: larval leg	*dpp*, *wg*, *rn*, *dac* and *Dll* are regulated by Hh and Lrp2	Gibson and Schubiger 1999; Mito et al. 2002; Nakamura et al. 2008a, b; Zhou et al. 2021
	Notch	blastema cell proliferation	*D. melanogaster*: wing disc	/	Blanco et al. 2010
	EGFR	patterning	*G. bimaculatus*: nymphal leg	Downstream of Wg/Wnt signaling and regulates *al* and *dac*	Nakamura et al. 2008a, b
Histone modifications (H3K27me3)	E(z)	patterning (prevent deformities)	*G. bimaculatus*: nymphal leg; *T. castaneum*: larval leg	Suppress *dac* expression	Hamada et al. 2015; Chou et al. 2019
	Utx	patterning (joint formation)	*G. bimaculatus*: nymphal leg	Promote *Egfr* expression	Hamada et al. 2015

## Physiological conditions controlling regeneration

Physiological factors, mainly refers to developmental and physiological status, the degree of amputation, and the species diversity of animals, are correlated with regeneration (Table 1). However, the question of how these physiological factors function in appendage regeneration has been largely ignored. The physiological conditions regulating the regeneration of insect appendages are described below.

### Developmental and life stages

The developmental- or life stage-dependent appendage regeneration differences has been demonstrated in many aspects. First, metamorphosis is a common developmental phenomena in insects, and the regeneration capacity to be lost after metamorphosis in both hemimetabolous and holometabolous insects(Seifert et al. 2012), a phenomenon similar to that observed in anurans, which are vertebrates undergoing metamorphosis. The reason for the existence of such a dramatic change in regeneration ability between pre- and postmetamorphic animals is puzzling. Interestingly, the silverfish, an ametabolous insect, continue to molt and grow after sexual or reproductive maturity, and its appendage regeneration capacity is not lost in adults. Even though the relationship between regeneration and growth has long been appreciated (Voit et al. 1985), the reason for the loss of life stage-associated regeneration in adult hemimetabolous and holometabolous insects is still elusive; it will be challenging to clarify the underlying mechanism.

Second, there is a strong relationship between the larval/nymphal development stage and regeneration capacity. In some holometabolous insects, such as *Helicoverpa armigera*, appendage regeneration capacity depends on the remaining molting cycles. Intact legs can be regenerated after emergence in adult moths when the larval legs are excised at the first or second instars. However, the regenerated legs become malformed or disappear in adults when the legs are excised at the third to sixth instars (Yang et al. 2016). A strong relationship also exists between nymphal instars and distal tibia regeneration ability in some hemimetabolous Orthopteran insects, such as *G. bimaculatus* and *Locusta migratoria*. In these two species, no fewer than three molts are needed for complete tibia and tarsus regeneration, which means that the regeneration process will not be completed if amputation occurs in relatively older instars (Bando et al. 2018) (Fig. 1B).

In addition, insects show another interesting phenomenon, regeneration ability switch, during certain instars of the larval/nymphal stage. It has been shown that the regeneration does not take place after a critical point in the molt cycle of cockroaches and many other insects, while this regeneration capacity will be regained in next molting cycle (O’Farrell and Stock 1953).

### Degree of amputation

Another strong factor that influence appendage regeneration is the degree of amputation, which determining the ultimate integrity and length of regenerated tissues. The insect leg usually consists of five main segments arranged along the proximodistal axis in the following order: coxa, trochanter, femur, tibia, and tarsus (Fig. 1). In *P. americana*, when progressively amputated all five segments (tarsus, tibia. femur, trochanter, and coxa) of the metathoracic limb, the ability to regenerate the missing limb was found to depend on trauma severity (Li et al. 2018). When amputations are performed proximal to the femur, tibia, or tarsus, new complete legs complete can be regenerated within a single molting cycle. When the trochanter or coxa is removed, normal regeneration is disrupted, which means that the trochanter and coxa are the two most important podites for cockroach leg regeneration (Li et al. 2018). Cockroach leg tarsus also includes five tarsomeres. In German cockroach, *Blattella germanica*, when the tarsus is amputated at or proximal to the 3rd tarsomere, four segmented tarsus will regenerate; when the leg is amputated distal to the 3rd tarsomere, five segmented tarsus will regenerate (Tanaka et al. 1992). Interestingly, there are two autotomy (self-cutting) sites distal to the trochanter and tibia sites in insects. When amputation is performed at sites other than these two autotomy sites, autotomy preferentially occurs at one of these two sites to optimize the length and shape integrity of the regenerated legs (Marzullo 2016).

In Orthopteran insects such as *G. bimaculatus* and *L. migratoria*, the leg regeneration ability is also highly dependent on the degree of amputation. When amputation is performed at the distal tibia site, integral new legs can be regenerated after multiple molts (Fig. 1B). However, the regeneration ability decreases sharply when amputation is performed at the trochanter or femur, and only a tiny amount of tissue can be restored post eclosion even amputation occurs in the first instar (Yang et al. 2016). Similar results have been found in Heteropterans such as *Rhodnius prolixus* and *Oncopeltus fasciatus*: when amputations are performed in the proximal part of the leg, only one segment (the terminal one) can be regenerated, but when amputations are performed distal to the tibia, three tarsal segments can be regenerated (Luscher 1948; Shaw and Bryant 1974). Regarding antenna regeneration, the regeneration of the antennae in Orthoptera and Phasmida is easily accomplished if part of the flagellum is removed, while regeneration seems to be more difficult when the cut affects the two basal components (Maruzzo et al. 2005; Urvoy 1970).

### Species diversity

The regeneration ability varies according to the orders and even subgroups in an order. Maruzzo and Bortolin defined a four-level standard for describing regeneration ability: absent (no regeneration), poor (fails to produce a structurally normal limb), good (produces a structurally normal limb under some specific conditions) and very good (produces a structurally normal limb under most conditions) (Minelli et al. 2013). Regeneration ability is absent to poor in Hemiptera, absent to good in Endopterygota, and very good in Blattodea, Mantodea, Phasmatodea, Zygentoma and other taxa (Maruzzo et al. 2005; Minelli et al. 2013). The difference in regeneration ability can also be reflected by the number of molts required for regeneration. Some insects with good regeneration ability, such as Blattodea, can complete the whole regeneration process in one molting cycle, while some insects with poor regeneration ability, such as Hemiptera, require several molting stages (Bell and Adiyodi 1982; Mito et al. 2002) (Fig. 1). There is abundant evidence that regeneration abilities are sometimes very different even among closely related insects. In beetles, larval leg seems not to regenerate in Hydrophilidae and Dytiscidae, but they do regenerate in other families, such as the tenebrionid *Tenebrio molitor*, the dynastid *Oryctes nasicornis*, and the cerambycid *Rhagium indagator* (Maruzzo et al. 2005).

To clarify the relationship between leg regeneration variation and species diversity, Suzuki and colleagues linked leg types with Waddington’s epigenetic landscape model (Suzuki et al. 2019; Waddington 2014). Three leg tissue types, direct-developing/differentiated legs, larval legs, and imaginal discs, were generalized according to the developmental stage of the legs (Suzuki et al. 2019). Directly developing nymphal legs in hemimetabolous insects develop adult-like morphologies during embryogenesis, and their postembryonic development primarily involves an increase in size without a significant change in shape. Larval legs are neither identical to embryonic legs nor completely differentiated in evolutionally inferior holometabolous insects and likely represent a partially differentiated state. Imaginal discs are internal precursors of appendages that form during embryogenesis, and the cells have been suggested to have stem cell-like properties (Suzuki et al. 2019). Waddington’s epigenetic landscape model was established to illustrate the various paths of cell differentiation (Waddington 2014). In this theory, different leg types with distinct starting points of cells at the time of leg ablation result in different types of regeneration. To date, taxonomic sampling for insect appendage regeneration is limited and inconsistent, which makes it difficult to clarify regeneration variation using standard phylogenetic methods, and more ideal insect models with stronger regeneration ability should be explored.

## Internal mechanisms underlying regeneration

Based on the well-established genetic resources available in model organisms, great progress has been made in understanding regeneration at the molecular level. Many signaling pathways and epigenetic modifications, which will be mentioned below, are required for appendage regeneration in insects (Table 2).

### Signaling pathways

#### ROS and MAPK signaling

One key goal in regenerative biology is to reveal the nature of the earliest signals that initiate repair and regeneration (Liu et al. 2021). The initiation of a regenerative response after damage is based on cell communication between injured cells and surrounding tissue. In regeneration, reactive oxygen species (ROS) are one of the earliest signals produced by the NADPH oxidase enzyme DUOX in injured cells, and ROS can activate Jun-N-terminal kinase (JNK) and p38 signaling in both apoptotic cells and neighboring surviving cells (Fan et al. 2014; Santabárbara-Ruiz et al. 2015). Apoptotic cells can stimulate neighboring surviving cells to undergo additional proliferation (Fan et al. 2014; Fogarty et al. 2016). JNK and p38 are conserved subfamilies of mitogen-activated protein kinases (MAPKs) that sense and elicit cellular responses to stress and tissue damage (Roux and Blenis 2004). During wing disc regeneration, apoptotic signal-regulating kinase 1 (Ask1) and Akt kinase (Akt) sense ROS in both dying and living cells to activate moderate phosphorylation levels of p38 and JNK (Esteban-Collado et al. 2021; Santabárbara-Ruiz et al. 2019). Damage-induced JNK signaling leads to the upregulation of the Duox-maturation factor NIP, which in turn promotes ROS generation in regenerating tissue (Khan et al. 2017). Additionally, a calcium flash activates DUOX and thus triggers the production of ROS as a damage cue (Razzell et al. 2013). The JNK signaling pathway is required for the formation of actin cables and filopodial extensions during wound healing and the cell death-induced regeneration of *Drosophila* wing imaginal discs (Bergantiños et al. 2010; Bosch et al. 2005; Mattila et al. 2005). Elevated phosphorylation levels of JNK play a role in the formation of regenerative blastocytes. JNK signaling can promote the re-entry of cells into a proliferative state, which is necessary for regenerative growth. Inhibition of JNK function can reduce the number of mitotically active cells in the blastema (Mattila et al. 2005). The phosphorylation of p38 is another early response to *Drosophila* wing disc damage that occurs alongside JNK signaling (Santabárbara-Ruiz et al. 2019). PI3K/Akt signaling is necessary for Ask1 to phosphorylate p38, and the phosphorylation of p38 during regeneration is nutrient sensitive. This finding distinguishes p38 and JNK function in the cells involved in tissue repair and regenerative growth (Esteban-Collado et al. 2021), but the interaction between JNK and p38 is still elusive. As mentioned above, studies on the roles of JNK and p38 in insect regeneration have mainly focused on *Drosophila* imaginal discs; the functions of these proteins in other insect appendages are less clear. In the black cutworm *Agrotis ypsilon*, the Ras/MAPK pathway is required for the regenerative growth of wing discs, and the level of di-phosphorylated extracellular regulated kinase (ERK) is obviously higher in ablated discs (Xu et al. 2021). ERK, which represents the third branch of the MAPK pathway, has also been reported to be essential for tissue regeneration in planaria and vertebrates (De Simone et al. 2021; Tasaki et al. 2011), while its function in insect appendage or imaginal disc regeneration warrants further attention.

#### ECM signaling

The degradation and remodeling of the extracellular matrix (ECM) is critically important for many cellular processes, such as wound healing, and these processes are mainly mediated by the family of matrix metalloproteinase (MMP) enzymes. MMPs are thought to play an important role in wound healing and tissue repair in pathological conditions such as injury or infection (Rohani and Parks 2015). In *Drosophila, Mmp1* and *Mmp2* are upregulated during imaginal disc regeneration (Klebes et al. 2005), but only Mmp1 has been found to control regeneration by regulating the cell cycle arrest of nonblastema cells during leg imaginal disc regeneration (McClure et al. 2008). In *Tribolium castaneum*, wound healing also requires the reconstruction of ECM. The knockdown of MMPs could delay wound healing, resulting in the disruption of subsequent blastema formation during larval leg regeneration (Mitten et al. 2012).

#### JAK/STAT signaling

Studies of Janus kinase/signal transducer and activator of transcription (JAK/STAT) signaling in *Drosophila* have revealed its multiple conserved roles in a wide range of biological processes, including immune responses, cell proliferation, and stem cell maintenance (Zeidler et al. 2000). The ligands involved in JAK/STAT signaling are secreted cytokines (Upd, Upd2, Upd3) in *Drosophila*. The cytokines bind to the interleukin receptor Domeless (Dome) and activate the nonreceptor tyrosine kinase Hopscotch (Hop, an ortholog of mammalian JAK), which phosphorylates the transcription factor STAT. Phosphorylated STAT homodimerizes and localizes to the nucleus to activate the transcription of target genes (Arbouzova and Zeidler 2006). JAK/STAT signaling also regulates the proliferation of blastema cells during *Drosophila* wing disc and cricket leg regeneration (Bando et al. 2013; La Fortezza et al. 2016), which suggests that JAK/STAT signaling is widely required during regeneration in insects. The JAK/STAT signaling pathway can be turned on to function in regeneration via the activation of the ROS/JNK/P38/Upd stress response cascade (Santabárbara-Ruiz et al. 2015). Further research has proven that the activation of the JAK/STAT signaling pathway can also be induced by the immunity-associated Toll signaling pathway, and the RNA interference (RNAi) knockdown of *Toll2* results in the downregulation of JAK/STAT signaling components and reduces blastema cell proliferation in regenerative cricket legs (Bando et al. 2022). The JAK/STAT signaling pathway synergistically regulates the proliferation of regenerated cells with other signaling pathways, such as Wg/Wnt signaling. It has also been reported that JAK/STAT signaling promotes cell survival in regenerating tissue (La Fortezza et al. 2016). In addition, the activated JAK/STAT pathway induces the paracrine factor *Drosophila* insulin-like peptide 8 (Dilp8) to delay the larval-to-pupal transition (Katsuyama et al. 2015).

#### TGF-β Family Signaling

The transforming growth factor-β (TGF-β) family signaling pathway is conserved in animals. Similar to the situation in mammals, the TGF-β family signaling pathway has two branches in insects, which are initiated by different ligands, activins and bone morphogenetic proteins (BMPs) (Parker et al. 2004). There are four ligands in the BMP signaling pathway, decapentaplegic (Dpp), Screw (Scw), Glass-bottom boat (Gbb), and Maverick (Mav), and their signals are transferred through the type II receptors Punt and Wishful thinking (Wit) and the type I receptors Thickveins (Tkv) and Saxophone (Sax). The transcription factor Smad1 (mothers against Dpp, Mad) is phosphorylated (pSmad) by type I receptors and binds to the common Smad (Co-Smad, Medea) to transduce all BMP signals (Upadhyay et al. 2017). In *G. bimaculatus*, the expression signal of *dpp* is localized in the dorsal zones of the blastema according to whole-mount in situ hybridization (Mito et al. 2002). The knockdown of *dpp* does not produce any detectable phenotype (Nakamura et al. 2008a, b). However, the knockdown of *Tkv* and *Mad* leads to a loss of tarsus regeneration ability in the most distal regions of regenerating leg segments and results in significantly elongated regenerated tibias along the proximodistal axis compared with tibias in normal legs, indicating that the canonical BMP pathway is involved in leg regeneration. Moreover, the BMP signaling pathway interacts with Dachsous/Fat signaling and *dachshund* (*dac*) to re-establish positional information and regulate the determination of leg size (Ishimaru et al. 2018). In *P. americana*, *dpp* and *Mad* are essential for leg regeneration, and knocking down either of these two genes can disrupt the regeneration process completely (Li et al. 2018). These findings suggest that the TGF-β family signaling pathway is involved in blastema formation and proximodistal axis remodeling. Since there are multiple ligands and receptors in the BMP signaling pathway subbranch and the more complex TGF-β family signaling pathway, the functions of these elements in regeneration warrant further attention.

#### Hippo signaling

The Hippo signaling pathway, also known as the Salvador-Warts-Hippo pathway, is an evolutionarily conserved network that plays a central role in regulating cell proliferation and cell fate to control organ growth and regeneration. In insects, the key components of Hippo signaling are Expanded (ex), Merlin (Mer), Hpo (Mst1/2), Sav (Sav1), Mats (Mob1/2) and Wts (Lats1/2). Hpo phosphorylates and activates Wts, which further phosphorylates Yorkie (Yki) to promote proteasome-mediated degradation. Yki promotes tissue growth by increasing the expression of positive regulators of cell growth and inhibitors of apoptosis together with other transcription factors, such as Scalloped (Sd). Thus, activation (or inactivation) of Hippo signaling suppresses (or promotes) cell proliferation via inhibition (or activation) of Yki activity. The ex and Mer are upstream inputs of Hpo and Wts kinases, while Sav and Mats are adaptor proteins of Hpo and Wts, respectively (Ma et al. 2019). In *Drosophila*, Yki is hyperactivated within wing discs in response to tissue damage whether the damage is induced surgically, genetically, or by irradiation. This Yki activation appears to be important for compensatory cell proliferation in wing disc regeneration, and the JNK signaling pathway acts upstream of the Hippo pathway (Grusche et al. 2011; Sun and Irvine 2011). The crucial role of Hippo signaling in leg regeneration has also been reported in crickets. In the early phase of regeneration, some Hippo signaling components are upregulated. This signaling is involved in blastema cell proliferation. The RNAi knockdown of *Wts*, *ex* and *Mer* lead to enlargement of the blastemal region and hyperproliferation of blastema cells in regenerating legs, and this phenotype can be reversed by *Yki* RNAi (Bando et al. 2018; Bando et al. 2009). These results suggest that *Yki* is essential for cell proliferation, while *Wts*, *ex* and *Mer* transcripts can influence the determination of leg size by inhibiting overproliferation during leg regeneration. Hippo signaling is also activated by Dachsous/Fat signaling to suppress cell proliferation during cricket leg regeneration (Bando et al. 2009).

#### Wg/Wnt signaling

The canonical Wnt signaling pathway proceeds through the binding of Wnt ligands to transmembrane cell surface receptors of the frizzled family. This binding initiates this signal transduction pathway that stabilizes the cytosolic transcription factor Armadillo (Arm, an ortholog of mammalian β-catenin), allowing Arm to translocate into the nucleus and activate the transcription of various genes (Bejsovec 2005; Widelitz 2005). In *Drosophila*, damage to the imaginal discs leads to ectopic expression of *Wingless* (*Wg*), the *Drosophila* ortholog of the secreted glycoprotein Wnt1, at the wound site, and *Wg* is crucial during regeneration after the cut injury and tissue ablation of leg and eye imaginal discs (Schubiger et al. 2010; Smith-Bolton et al. 2009; Sustar et al. 2011). A 3' enhancer controls *Wg* expression in the blastema of injured leg dics, and the *Wg* gene can be activated by JNK signaling in wing dics. Similar to *Drosophila* imaginal disc regeneration, *Wnt-1* and *Arm-2* knockdown prevents leg, antenna and maxilla regeneration in *T. castaneum* (Shah et al. 2011). In *G. bimaculatus*, *Wg* is expressed on the ventral side of the anteroposterior boundary after tibia amputation; during intercalary regeneration, the expression of *Wg* is induced in the proximal amputated region but not in the distal grafted donor (Mito et al. 2002). Reducing Wg/Wnt signaling via the knockdown of the signal transducer *Arm* blocks regeneration completely, while RNAi targeting *Wg* has no effect, possibly because of redundancy or incomplete knockdown; these findings indicate that the canonical Wg/Wnt pathway may be involved in the initiation of regeneration and in determination of positional information during leg regeneration (Nakamura et al. 2008a, b; Nakamura et al. 2007).

#### Hedgehog signaling

The Hedgehog (Hh) signaling pathway constitutes a small family of secreted Hh proteins that together regulate multiple aspects of animal development, tissue homeostasis and regeneration (Ingham 2022). This signaling pathway was originally discovered and well characterized in *Drosophila* (Ingham et al. 2011). In the presence of the *Hh* ligand, the phosphorylation of Smoothened (Smo) promotes the Fused-mediated phosphorylation of the scaffold protein Costal 2 (Cos2) and Suppressor of fused (Sufu). Then, the transcription factor Cubitus interruptus (Ci) separates from Sufu and Cos2 and translocates into the nucleus to activate Hh target genes (Villarreal et al. 2015). In *Drosophila*, Hh directs anterior/posterior conversion by activating the posterior-specific transcription factor *engrailed* (*en*) to regulate anterior cells during leg disc regeneration. In the absence of Hh activity, prothoracic leg disc fragments fail to undergo anterior/posterior conversion but can still regenerate missing anterior pattern elements (Gibson and Schubiger 1999). Hh is responsible for regulating the posterior region of the regenerated leg and affects leg patterning together with Dpp and Wg in crickets (Mito et al. 2002); moreover, Hh RNAi results in altered P/D patterning and supernumerary axis formation (Nakamura et al. 2008a, b). In *Harmonia axyridis*, Hh and its auxiliary receptor, Lrp2, are required for the proper patterning and growth of the regenerative leg; the targets of canonical Hh signaling are required for regenerative growth, which contributes to leg length but is not essential for pattern formation in the regenerative leg. In addition, *dpp*, *wg*, *rn*, *dac* and *Dll* are regulated by Hh and Lrp2 during leg regenerative patterning in *H. axyridis* (Zhou et al. 2021).

#### Notch signaling

Notch signaling has a simple framework that is highly conserved throughout the animal kingdom. The Delta and Serrate ligands and their receptor, Notch, are all transmembrane proteins. Ligand binding promotes two proteolytic cleavage events in the Notch receptor and releases the Notch intracellular domain (Nicd), which then translocates to the nucleus and cooperates with the DNA-binding protein CSL (Su(H)) and its coactivator Mastermind (Mam) to promote the expression of target genes (Bray 2006). Notch signal transduction involves wound healing, tissue repair, regeneration and stem cell renewal (Artavanis-Tsakonas et al. 1995). With regard to insect regeneration, the Notch (N) receptor and its mediator enhancer of split (E(spl)) are differentially expressed in regenerating wing discs and are important for blastema proliferation in *Drosophila* (Blanco et al. 2010). Notch signaling is also activated during leg regeneration in *H. axyridis* (Zhou et al. 2021). Although Notch signaling has been reported to be involved in zebrafish fin and heart regeneration (Grotek et al. 2013; Raya et al. 2003), tadpole tail regeneration (Beck et al. 2003), and adult newt retinal regeneration (Nakamura and Chiba 2007), whether this signaling directly contributes to leg regeneration in insects is unclear, and more attention should be given to this topic.

#### Other signaling

Other signaling pathways, such as the epidermal growth factor receptor (EGFR) and fibroblast growth factor receptor (FGFR) signaling pathways, are also involved in regeneration. EGFR signaling is required for distal leg patterning in regeneration during the nymphal stage in crickets. EGFR signaling acts downstream of canonical Wg/Wnt signaling and regulates the appendage proximodistal patterning genes *aristaless* (*al*) and *dac* during distal leg regeneration (Nakamura et al. 2008a, b). However, even though FGF is required for regeneration in vertebrates (Satoh et al. 2011), the silencing of the FGF receptor does not interfere with the initial stages of leg regeneration in *T. castaneum* (Mitten et al. 2012), which suggests that the role of FGF in leg regeneration might be unique to vertebrates.

### Epigenetic modifications

Epigenetic modifications provide an additional layer of genome regulation and participate in the control of many fundamental processes, such as development and regeneration. DNA methylation on cytosine (5-methylcytosine, 5mC) is the most abundant DNA modification in animals, but functional research on 5mC modification in insect appendage regeneration is scarce. Multiple posttranslational protein modifications, such as methylation, acetylation, phosphorylation and ubiquitylation, can be established on specific amino acid residues of histones. Polycomb group (PcG) proteins are involved in stabilizing chromatin structure through histone modification and act as transcriptional repressors (Aloia et al. 2013; Schuettengruber et al. 2017). More than a dozen PcG proteins can be divided into three separate multiprotein complexes: Polycomb repressive complex 1 (PRC1), Polycomb repressive complex 2 (PRC2) and Pho repressive complex (PhoRC). Enhancer of zeste (E(z)) and Pc belong to PRC2 and PRC1, respectively. E(z) and ubiquitously transcribed tetratricopeptide repeat gene on the X chromosome (Utx), which regulate the methylation and demethylation, respectively, of histone H3 lysine 27 (H3K27me3). In crickets. E(z) regulates the rearrangement of the regenerative leg by suppressing *dac* expression to prevent deformities, such as the formation of an extra leg segment between the tibia and tarsus, and *E(z)* RNAi produces an extra tibia segment; Utx participates in tarsus joint formation during leg regeneration by regulating Egfr expression (Hamada et al. 2015). The knockdown of *E(z)* and *Pc* expression inhibits leg regeneration after ablation at the coxa in *T. castaneum* (Chou et al. 2019). *E(z)* knockdown in *T. castaneum* leads to a loss of regenerative ability, which differs from that in cricket nymphal legs. This curious difference may arise from the different amputation sites and different degrees of differentiation involved. In *Drosophila* imaginal discs, regeneration is coupled to the regulation of PcG. The downregulation of PcG function is observed in proliferating regenerative cells, and the activation of JNK leads to the downregulation of PcG genes (Lee et al. 2005). Since the types of epigenetic modifications are numerous, more work on other modifications is warranted to explore their functions during insect regeneration.

## Conclusions and perspectives

The three main factors (developmental and life stages, amputation degree and species diversity) mentioned above are key factors related to physiology and have received relatively more attention than other factors in the insect appendage regeneration field (Table 1). Although several interesting findings in this context have been reported, rigorous experimental studies have largely been ignored in this field due to the difficulty of separating physiological factors from each other; hence, related studies will greatly promote the elucidation of the relevant mechanisms. Notably, appendage regeneration capacity varies among insects, and the identification of better or even ideal regenerative models is important to promote research in this field (Suzuki et al. 2019). In addition, it will be challenging to address the reasons for regenerative diversity among insects (such as that between holometabolous and hemimetabolous insects), but the application of a comparative evolutionary developmental biology approach to study limb regeneration in a variety of insect species is a good option. In addition, a prerequisite for insect appendage regeneration is a healthy growth status, and other physiology-associated environmental factors, such as nutritional condition, water supply, temperature and humidity, might play key roles in regeneration. Thus, more attempts to clarify the function of physiological conditions in insect appendage regeneration are warranted.

The internal mechanisms underlying appendage regeneration in insects are complicated; many of the signaling pathways and epigenetic modifications mentioned above are involved in different regeneration processes (Table 2). There are also complicated interactions among signaling pathways and among epigenetic modifications. Successful regeneration cannot be achieved when even one of these factors is disrupted. However, studies on the specific roles of internal factors in regeneration processes are limited in insects, and the available studies on interactions among these signaling pathways and epigenetic modifications during different regeneration processes are insufficient; hence, further investigations are needed in the future. In addition to the signaling pathways and epigenetic modifications discussed above, other signaling pathways or epigenetic modifications that have received less attention might also contribute to appendage regeneration in insects, and relevant efforts and breakthroughs will greatly promote progress in this field.

As mentioned above, both physiological factors and internal mechanisms play important roles in appendage regeneration in insects, while the interaction between physiological factors and internal mechanisms, such as signaling pathways, is unclear. Thus, the combined study of physiological and internal factors will be an attractive research direction in the insect appendage regeneration field. In conclusion, the advantages conferred by recent advances in molecular techniques such as CRISPR-based gene knockout and knock-in strategies, high-throughput sequencing methods (RNA-seq, single-cell RNA-seq, spatial transcriptome RNA-seq), and high-sensitivity tissue or cell staining techniques will allow us to delve into the molecular and cellular mechanisms of limb regeneration across a variety of species. We believe that the opportunity to solve the mystery of limb regeneration will come in the near future.

## Data Availability

Not applicable.

## Abbreviations

ROS: Reactive oxygen species
MAPKs: Mitogen-activated protein kinases
JNK: Jun-N-terminal kinase
Ask1: Apoptotic signal-regulating kinase 1
ERK: Extracellular regulated kinase
ECM: Extracellular matrix
MMP: Matrix metalloproteinase
JAK/STAT: Janus kinase/signal transducer and activator of transcription
Dilp8: *Drosophila* Insulin-like peptide 8
TGF-β: Transforming growth factor-β
BMPs: Bone morphogenetic proteins
EGFR: Epidermal growth factor receptor
FGFR: Fibroblast growth factor receptor
PRC1: Polycomb repressive complex 1
PRC2: Polycomb repressive complex 2
PhoRC: Pho repressive complex
E(z): Enhancer of zeste
PcG: Polycomb group
